# Association between family health and fertility intention among women of childbearing age in China: evidence from a large multi-stage cross-sectional survey

**DOI:** 10.7189/jogh.16.04165

**Published:** 2026-07-10

**Authors:** Xiaojun Tan, Jie Hua, Shurong Huang, Simei Zhou, Wenzhi Cai, Ling Chen

**Affiliations:** 1 Department of Gastrointestinal Surgery, Shenzhen Hospital, Southern Medical University, Shenzhen, Guangdong, China; 2 Department of Nursing, Shenzhen Hospital, Southern Medical University, Shenzhen, China; 3 School of Nursing, Southern Medical University, Guangzhou, China; 4 Department of Obstetrics and Gynaecology, Shenzhen Hospital, Southern Medical University, Shenzhen, China; 5 Department of Obstetrics, Shenzhen LongHua Maternity and Child Healthcare Hospital, Shenzhen, China

## Abstract

**Background:**

Fertility has consistently been an imperative issue worldwide. Fertility intention plays a crucial role in predicting individuals’ actual fertility behaviour. Family health may be effective in improving women’s fertility intentions. However, the relationship between family health and fertility intention remains unclear. Therefore, we aimed to examine whether family health was associated with fertility intention among Chinese women of childbearing age.

**Methods:**

Using data from Psychology and Behaviour Investigation of Chinese Residents in 2021, we conducted a nationwide cross-sectional study in 1405 eligible women. Family health was measured using the Short Form of the Family Health Scale. Fertility intention was measured using a self-made questionnaire. We applied multivariate logistic regression and restricted cubic spline models to examine the association and dose-response relationship between family health and fertility intention. Subgroup analyses and interaction tests were further used to examine differences across strata.

**Results:**

After full adjustment, higher family health was associated with greater fertility intention (odds ratio (OR) = 1.07; 95% confidence interval (CI) = 1.03, 1.10), with consistent findings across quartiles. A linear dose-response relationship was observed (*P* for nonlinear = 0.724). Significant interactions by age and number of children were identified (*P* for interaction <0.001), with stronger associations among women aged 19–40 years without children.

**Conclusions:**

Family health is an important determinant of fertility intention, particularly among younger women without children. Therefore, public health policies aiming at enhancing fertility intentions should consider the importance of improving family health.

The decline in global fertility rates has garnered significant attention. From 2000 to 2020, the global total fertility rate decreased from 2.58 to 2.31 children per woman [1]. This decline is expected to continue, reaching 1.83 by 2050 and 1.59 by 2100 [2]. Such sustained low fertility poses challenges, including population ageing, workforce decline, and economic slowdown [2,3]. In China, the decrease has been even more dramatic, with the total fertility rate dropping from 1.22 in 2000 to 1.07 in 2022 [1]. Since the implementation of the one-child policy in 1979, China’s fertility rate has remained below the replacement level [4]. In response, China gradually relaxed birth policies by introducing selective and universal two-child policies [5] followed by a three-child policy [6]. Despite these efforts, the number of births continued to decline, reaching 9.02 million by 2023 [7]. As the effects of the newly implemented population policy wane and the number of women of childbearing age decreases, there is a growing risk of a continued decline in the birth rate [8]. In this context, understanding the determinants of fertility intentions among women of childbearing age is critical for policymakers and researchers.

Fertility intention, defined as an individual’s desire to have children in the future, is a robust predictor of actual fertility behaviour [9]. Existing research generally categorises the determinants of fertility intention into three major dimensions: economic conditions, cultural expectations, and gender norms. Among these, economic factors constitute the most rigid and immediate constraints on fertility intention. Rising housing prices [10], increasing childcare expenses [11], and the potential income losses women may incur due to childbearing [12] collectively suppress fertility desire. In China, the disproportionately high share of educational expenditure has emerged as a particularly salient barrier. Combined with intense competition within the education system, this has generated a distinct form of education-related anxiety that further discourages childbearing [13]. By contrast, across Organisation for Economic Co-operation and Development countries, net childcare costs account for only 11% of the average parental wage, indicating a comparatively light financial burden [14]. Cultural expectations influence fertility intention through two parallel pathways: traditional norms and modern value orientations. Within the context of traditional Chinese culture, residual beliefs emphasising lineage continuation and gender preference continue to exert influence among certain groups [15] However, with the expansion of education and the diffusion of individualism, childbearing is increasingly framed as a personal choice rather than a familial obligation, contributing to a decline in the ideal number of children [16]. Gender norms further shape fertility intention by structuring the social support system surrounding childbearing. The absence of strong normative expectations for male participation in childcare places a disproportionate caregiving burden on women, thereby reducing their willingness to have children [10]. Concurrently, the implicit motherhood penalty prevalent in the Chinese labour market intensifies women’s concerns about career interruption and long-term income loss [12]. Prior research suggests that socio-cultural pressure represents the most significant driver of negative fertility intention in China [10], in sharp contrast to Nordic countries, where higher levels of gender equality have been institutionalised [17]. Ultimately, these macro-level and individual-level factors converge within the household. Economic conditions shape a family’s capacity to bear the costs of raising children; improvements in the social security system have weakened the traditional reliance on offspring for old-age support, thereby reducing the perceived necessity of childbearing; and gender norms directly influence the division of domestic labour, exacerbating women’s difficulties in balancing career development and childcare responsibilities. However, family-level factors, which play a central role in individual decision-making, have received limited attention. This gap is particularly problematic because fertility decisions are fundamentally made within family contexts, where individual preferences, resources, and constraints interact in complex ways. However, family-level factors, which play a central role in individual decision-making, have received limited attention. This gap is particularly problematic because fertility decisions are fundamentally made within family contexts, where individual preferences, resources, and constraints interact in complex ways.

Family health refers to the overall well-being formed by individual health statuses, interpersonal relationships, and shared family resources [18]. Its assessment requires a multidimensional approach that considers factors such as family functioning, economic status, social networks, emotional support, and access to external resources [19]. Drawing on family systems theory [20] and ecological models of human development [21], this study conceptualises family health as a key explanatory construct for fertility intentions. Prior research has largely focused on individual-level determinants, such as income, education, and social support; however, such atomistic approaches overlook how these factors interact dynamically within family systems. Family health, as measured by the Family Health Scale-Short Form (FHS-SF) [19], is a higher-order construct that captures the emergent properties of family functioning across four interrelated dimensions: social-emotional processes, healthy lifestyles, resources, and external supports. The theoretical contribution of family health lies in three aspects. First, it captures interaction effects that individual-level analyses fail to detect, as the influence of factors such as economic resources depends on the broader family emotional and relational context. Second, family health reflects the family’s collective capacity to mobilise and integrate resources toward shared goals, emphasising that fertility decisions arise from family-level negotiations, resource allocation, and shared future orientations rather than isolated individual attributes. Third, family health functions as a mediating construct linking macro-level contexts – such as economic conditions, cultural norms, and policy environments – to individual fertility intentions. Through this lens, broader social forces are translated into concrete family environments that either facilitate or constrain childbearing. Consequently, adopting a family health approach enables a theoretically grounded understanding of fertility intentions that goes beyond fragmented individual-level explanations.

This study aims to integrate and elevate fragmented influencing factors into the multi-dimensional, comprehensive influencing factor of ‘family health,’ and to explore the relationship between family health, as an independent core influencing factor, and fertility intention. We hypothesised that family health may be positively associated with fertility intention. By exploring this relationship, we aimed to provide new insights into the factors that shape fertility intentions and to inform policies and interventions that promote healthy families and support women's reproductive choices.

## METHODS

### Survey design and data collection

We employed publicly available data from the Psychology and Behaviour Investigation of Chinese Residents (PBICR), a nationwide cross-sectional survey conducted from 10 July to 15 September 2021, using multistage random sampling. First, 31 provincial capital cities, including five autonomous regions and four municipalities (including Beijing, Tianjin, Shanghai, and Chongqing), were directly included. Second, two to six prefecture-level cities were selected using a random number table, and, finally, a total of 120 cities (excluding Hong Kong, Macao, and Taiwan) were included.

Within the PBICR survey, investigators underwent comprehensive training in sampling methods, research tools, and quality control. The targeted participants had to be aged >12 years, able to understand each questionnaire item, and able to complete it independently or with the help of an investigator. People who were confused, were experiencing mental health difficulties or cognitive impairment, or were unwilling to participate in the survey were excluded. After confirming that the respondent meets the inclusion criteria, investigators used the Online Questionnaire Star platform (Changsha Ranxing Information Technology Co., Ltd, Changsha, China) to distribute questionnaires, obtain informed consent, and record questionnaire numbers. For participants with cognitive capacity but physical inability to complete the questionnaire, the investigators conducted one-on-one interviews and completed the questionnaire for them instead.

During the 2021 PBICR survey’s data processing phase, two researchers were designated to perform logical checks. Questionnaires that did not meet the predetermined screening criteria were excluded, ensuring the quality and reliability of the data. In the end, a total of 11 709 questionnaires were distributed, and 11 031 valid questionnaires were returned, resulting in 94.2% response rate.

### Study participants

**Figure 1:**
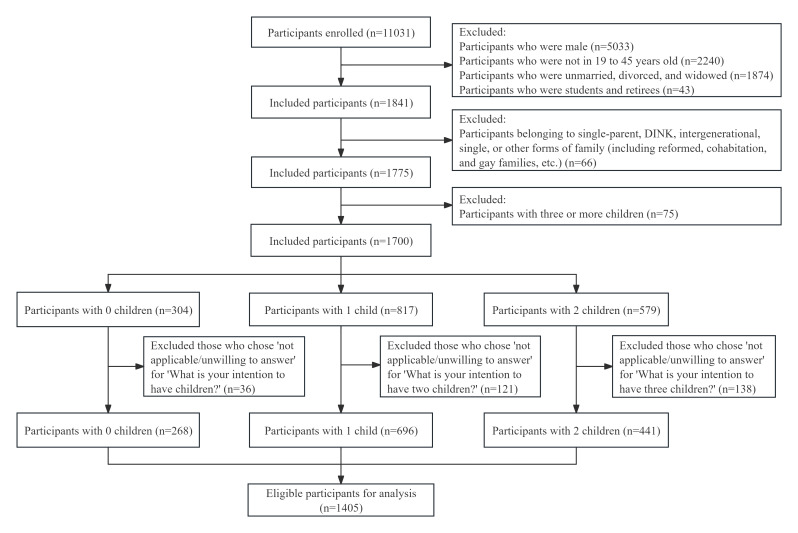
Flowchart of participant enrolment. ‘Single-parent family’ refers to a family consisting of divorced, widowed, or unmarried single fathers or mothers and their children or adopted children; ‘Dink family’ refers to a voluntarily infertile family consisting of a couple; ‘Intergenerational family’ refers to a family with only grandparents and grandchildren, where the parents have left the family for some reasons; ‘Single family’ refers to individuals who are not married at the marriageable age or have not remarried after divorce but live alone.

The sample screening process began with 11 031 participants (**Figure 1**). Since this study focuses on the association between family health and fertility intention among women of childbearing age, the analytic sample was screened as follows: restriction to female participants; restriction to women aged 19–45 years; exclusion of unmarried, divorced, or widowed women; exclusion of women identified as students or retirees; exclusion of participants from non-two-parent family structures, including single-parent, dual income, no kids, intergenerational, single-person, or other non-traditional family forms (*e.g.* reconstituted, cohabiting, or same-sex families); and exclusion of questionnaires with missing data on key variables or invalid responses. As specified in point six, the missing rates for each group are as follows: participants with no children, 11.84% (n/N = 36/304); participants with one child, 14.81% (n/N = 121/817); and participants with two children, 23.83% (n/N = 138/579). After this screening process, the final analytic data set comprised 1405 valid questionnaires. This study is reported in line with the STROBE checklist [22].

The missing data resulted from participants selecting the ‘unwilling to answer’ option in self-reports, which is missing not at random. Despite this, the sample size remains sufficiently large to meet the statistical power requirements for subsequent analyses. Furthermore, after excluding samples with missing values, the distribution of basic characteristics across core subgroups did not change significantly, thereby avoiding potential bias from missing-value imputation.

### Measures

#### Fertility intention

Fertility intention was measured using a self-designed questionnaire consisting of three questions tailored to women based on their number of children: ‘What is your intention to have children?’ for those without children, ‘What is your intention to have two children?’ for those with one child, and ‘What is your intention to have three children?’ for those with two children. Response options for each question were ‘no intention at all,’ ‘no intention,’ ‘normal,’ ‘with intention,’ and ‘with a strong intention.’ The five responses are assigned scores ranging from one to five, where a score of one represented ‘no intention at all’ and a score of five denoted ‘with a strong intention.’ Scores from one to three were categorised as the low fertility intention group, whereas scores from four to five were classified as the high fertility intention group.

#### Family health

Family health was measured using the FHS-SF [19]. The scale comprises 10 items across four dimensions: family social and emotional health processes, family healthy lifestyle, family health resources, and family external social supports. Each item is rated on a five-point Likert scale from one (strongly disagree) to five (strongly agree), with items six, nine, and 10 reverse-scored. Total score ranges from 10–50, with higher scores indicating better family health. The Chinese version of the FHS-SF has demonstrated good validity and reliability [23]. In this study, Cronbach’s alpha for the FHS-SF was 0.84.

#### Covariates

Covariates were chosen based on prior research [24–26]. Possible relevant covariates included age, ethnicity, highest educational level, region, place of residence, current occupational status, family type, family per capita monthly income, medical insurance, number of children, depression, anxiety, perceived social support, and pressure.

Depressive symptoms were measured using the Patient Health Questionnaire-9 (PHQ-9) [27]. Each item is scored from zero (not at all) to three (nearly every day), yielding a total score ranging from zero to 27, with higher scores indicating more severe depressive symptoms. The Chinese version has demonstrated good reliability and validity in general populations [28]. In this study, Cronbach’s alpha for the PHQ-9 was 0.93.

Anxiety symptoms were assessed using the Generalised Anxiety Disorder-7 (GAD-7) [29]. Each item is scored from zero (not at all) to three (nearly every day), yielding a total score of zero to 21, with higher scores indicating more severe anxiety symptoms. The Chinese version of GAD-7 has been validated across various Chinese populations [30,31]. In this study, Cronbach’s alpha for the GAD-7 was 0.95.

Perceived social support was measured using the Multidimensional Scale of Perceived Social Support (MSPSS) [32]. The scale comprises 12 items across three dimensions: family support, friend support, and significant others support. Each item is rated on a seven-point scale from one (strongly disagree) to seven (strongly agree). Total scores range from 12–84, with higher scores indicating greater perceived social support. The Chinese version of the MSPSS has been validated in various Chinese groups [33–35]. In this study, Cronbach’s alpha for the MSPSS was 0.96.

Pressure was measured using a self-designed questionnaire comprising three items: ‘How would you rate your ability to cope with stress?’ ‘How would you rate the stress levels in your life (at home and at work) over the past two weeks?’ and ‘How would you rate the stress levels in your life (at home and at work) over the past year?’ Respondents rated each item on a scale from one to six to indicate their stress levels. Total scores range from three to 18, with higher scores reflecting greater pressure. In this study, Cronbach’s alpha for the questionnaire was 0.86.

### Statistical analyses

Associations between family health (overall score and four dimensions) and fertility intention were examined using multivariable logistic regression models, with odds ratios (ORs) and 95% confidence intervals (CIs) reported. The crude model (model 1) did not adjust for any covariates. Model 2 was adjusted for inherent demographic factors, including age and ethnicity. Model 3 was adjusted for all covariates. To validate the association between family health and fertility intention, the continuous family health variable was categorised into quartiles for analysis. Besides, the restricted cubic spline (RCS) model was constructed to explore the dose-response relationship between family health and fertility intention.

To assess robustness, sensitivity analyses were conducted by modelling family health as both a continuous variable and a quartile-based categorical variable. Restricted cubic spline models were further applied to examine potential nonlinear dose-response relationships. The direction and magnitude of associations remained largely consistent across alternative model specifications and levels of covariate adjustment.

To examine potential heterogeneity in the association between family health and fertility intention, we conducted subgroup analyses stratified by key demographic characteristics (*e.g.* age, income). These stratification variables were selected a priori based on their theoretical relevance; however, given the number of interaction terms tested, these analyses are considered exploratory.

All statistical analyses were conducted in *R*, version 4.3.1 (R Core Team, Vienna, Austria). Two-sided *P*-value <0.05 was considered statistically significant.

## RESULTS

### Characteristics of the study population

A total of 1405 participants were analysed, and 17.65% reported high fertility intention (**Table 1**). Compared with those with low fertility intention, participants with high fertility intention tended to be younger, have higher educational attainment, be employed, be childless, and report higher levels of anxiety and perceived social support (*P* < 0.05).

**Table 1:** Characteristics for the study population by fertility intention*

	Total (n = 1405)	Fertility intention		χ2/t-test	*P*-value
**Characteristics**		**Low intention (n = 1157)**	**High intention (n = 248)**		
Age group in years				96.956	<0.001
*19–30*	328 (23.35)	217 (18.76)	111 (44.76)		
*31–40*	592 (42.14)	489 (42.26)	103 (41.53)		
*41–45*	485 (34.52)	451 (38.98)	34 (13.71)		
Ethnicity				0.959	0.328
*Han*	1337 (95.16)	1098 (94.90)	239 (96.37)		
*Minority*	68 (4.84)	59 (5.10)	9 (3.63)		
Highest educational level				27.527	<0.001
*Junior high or below*	283 (20.14)	258 (22.30)	25 (10.08)		
*Senior high or specialty education*	465 (33.10)	391 (33.79)	74 (29.84)		
*Undergraduate or above*	657 (46.76)	508 (43.91)	149 (60.08)		
Region				0.025	0.988
*Eastern China*	734 (52.24)	604 (52.20)	130 (52.42)		
*Central China*	385 (27.40)	318 (27.48)	67 (27.02)		
*Western China*	286 (20.36)	235 (20.31)	51 (20.56)		
Place of residence				0.122	0.727
*Rural*	312 (22.21)	259 (22.39)	53 (21.37)		
*Urban*	1093 (77.79)	898 (77.61)	195 (78.63)		
Current occupational status				5.790	0.016
*Incumbency*	1036 (73.74)	838 (72.43)	198 (79.84)		
*No fixed occupation*	369 (26.26)	319 (27.57)	50 (20.16)		
Family type†				2.566	0.109
*Couple or core family*	1191 (84.77)	989 (85.48)	202 (81.45)		
*Stem or joint family*	214 (15.23)	168 (14.52)	46 (18.55)		
Family per capita monthly income in CNY				2.882	0.237
*≤3000*	316 (22.49)	265 (22.90)	51 (20.56)		
*3001–6000*	610 (43.42)	509 (43.99)	101 (40.73)		
*>6000*	479 (34.09)	383 (33.10)	96 (38.71)		
Have medical insurance				0.007	0.932
*Yes*	1210 (86.12)	996 (86.08)	214 (86.29)		
*No*	195 (13.88)	161 (13.92)	34 (13.71)		
Number of children				169.603	<0.001
*0*	268 (19.07)	149 (12.88)	119 (47.98)		
*1*	696 (49.54)	601 (51.94)	95 (38.31)		
*2*	441 (31.39)	407 (35.18)	34 (13.71)		
Depression scores, x̄ (SD)	6.03 (5.10)	5.94 (4.91)	6.46 (5.89)	–1.292	0.197
Anxiety scores, x̄ (SD)	4.41 (4.32)	4.27 (4.13)	5.06 (5.08)	–2.288	0.023
Perceived social support scores, x̄ (SD)	61.56 (12.01)	61.11 (12.04)	63.67 (11.70)	–3.053	0.002
Self-rated pressure scores, x̄ (SD)	9.65 (3.74)	9.62 (3.72)	9.79 (3.84)	–0.630	0.529

CNY – Chinese Yuan, SD – standard deviation, x̄ – mean, χ^2^ – χ^2^ test

*Presented as n (%) unless specified otherwise.

**†**Couple family refers to a family consisting of only a husband and wife; Core family refers to a family composed of parents and unmarried children; Stem family refers to a family consisting of parents and married children; Joint family means a family comprising parents and two or more married children, or siblings who remain undivided after marriage.

### Family health levels

The high-fertility-intention group had a significantly higher average family health score than the low-fertility-intention group (*P* = 0.002) (**Table 2**). Specifically, the high fertility intention group scored higher in family social and emotional processes, family healthy lifestyle, and family external social support (*P* < 0.05). No significant difference was found in family health resources between the groups (*P* = 0.354).

**Table 2:** Short Form of the Family Health Scale Scores for participants, x̄ (SD)

	Total (n = 1405)	Fertility intention		t-test	*P-*value
**Items**		**Low intention (n = 1157)**	**High intention (n = 248)**		
Item 1	4.09 (0.87)	4.05 (0.89)	4.25 (0.77)	–3.290	0.001
Item 2	4.04 (0.93)	4.01 (0.94)	4.15 (0.88)	–2.152	0.032
Item 3	4.13 (0.87)	4.10 (0.88)	4.31 (0.80)	–3.557	<0.001
Item 4	4.09 (0.87)	4.07 (0.88)	4.18 (0.82)	–1.893	0.059
Item 5	4.13 (0.84)	4.10 (0.86)	4.27 (0.77)	–2.972	0.003
Item 6	3.84 (1.20)	3.82 (1.19)	3.92 (1.25)	–1.190	0.234
Item 7	3.85 (0.85)	3.82 (0.86)	4.00 (0.81)	–3.214	0.001
Item 8	3.98 (0.95)	3.96 (0.95)	4.08 (0.91)	–1.880	0.060
Item 9	3.20 (1.23)	3.19 (1.22)	3.24 (1.26)	–0.591	0.555
Item 10	3.52 (1.22)	3.51 (1.21)	3.55 (1.27)	–0.507	0.612
Family social and emotional health processes	12.25 (2.43)	12.16 (2.46)	12.68 (2.24)	–3.035	0.002
Family healthy lifestyle	8.22 (1.63)	8.16 (1.65)	8.49 (1.53)	–2.907	0.004
Family health resources	10.55 (3.00)	10.52 (2.97)	10.71 (3.11)	–0.926	0.354
Family external social supports	7.83 (1.60)	7.78 (1.60)	8.08 (1.55)	–2.769	0.006
Total scores	38.86 (6.37)	38.62 (6.36)	39.97 (6.35)	–3.033	0.002

### Associations between family health and fertility intention

**Figure 2:**
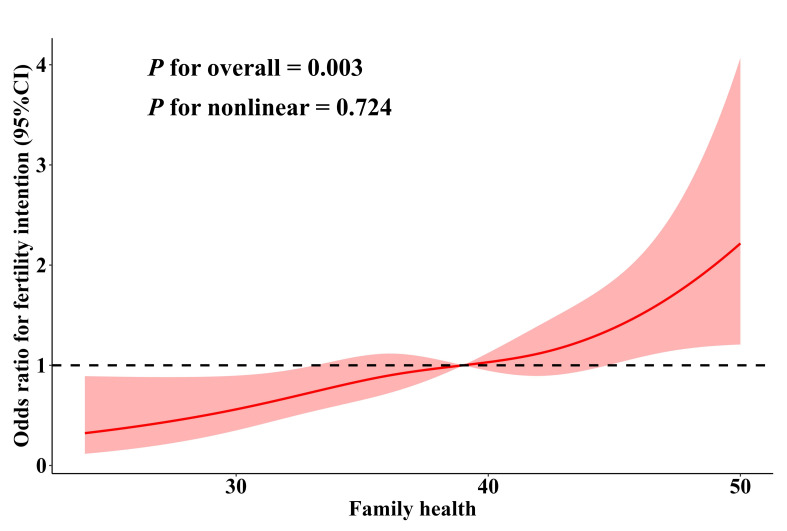
Restricted cubic spline for the association between family health and fertility intention, with all covariates controlled. CI – confidence interval.

Higher levels of family health were associated with greater fertility intention, and this association remained stable after successive covariate adjustments (**Table 3**). In the fully adjusted model, each incremental increase in family health score was associated with a higher likelihood of fertility intention, with an OR of 1.07. When family health was categorised, women in the highest quartile exhibited substantially higher fertility intention compared with those in the lowest quartile (OR = 2.67), indicating a pronounced gradient effect. Consistent with these findings, the RCS model revealed an approximately linear increase in fertility intention with improving family health (**Figure 2**). Across dimensions, most aspects of family health showed positive associations with fertility intention, whereas family health resources demonstrated a weaker relationship (Table S1 in the **Online Supplementary Document**).

**Table 3:** The association between family health and fertility intention explored by logistic regression

Family health (scores)	Model 1, OR (95% CI)*	*P*-value	Model 2, OR (95% CI)†	*P*-value	Model 3, OR (95% CI)‡	*P*-value
Continuous	1.03 (1.01, 1.06)	0.003	1.04 (1.02, 1.07)	<0.001	1.07 (1.03, 1.10)	<0.001
Categories						
*Quartile 1 (≤34)*	Ref.		Ref.		Ref.	
*Quartile 2 (34–39)*	1.28 (0.86, 1.90)	0.220	1.30 (0.86, 1.96)	0.209	1.54 (0.98, 2.43)	0.064
*Quartile 3 (40–44)*	1.29 (0.87, 1.92)	0.200	1.36 (0.91, 2.05)	0.137	1.70 (1.03, 2.83)	0.040
*Quartile 4 (>44)*	1.74 (1.19, 2.55)	0.004	1.94 (1.30, 2.89)	0.001	2.67 (1.53, 4.68)	<0.001

CI – confidence interval, OR – odds ratio, ref – reference

*No covariates were adjusted.

**†**Adjusted for inherent demographic factors including age and ethnicity.

‡Adjusted for all covariates, including age, ethnicity, highest education level, region, place of residence, current occupational status, family type, family per capita monthly income, having medical insurance, number of children, depression, anxiety, perceived social support, and self-rated pressure.

### Subgroup analyses

**Figure 3:**
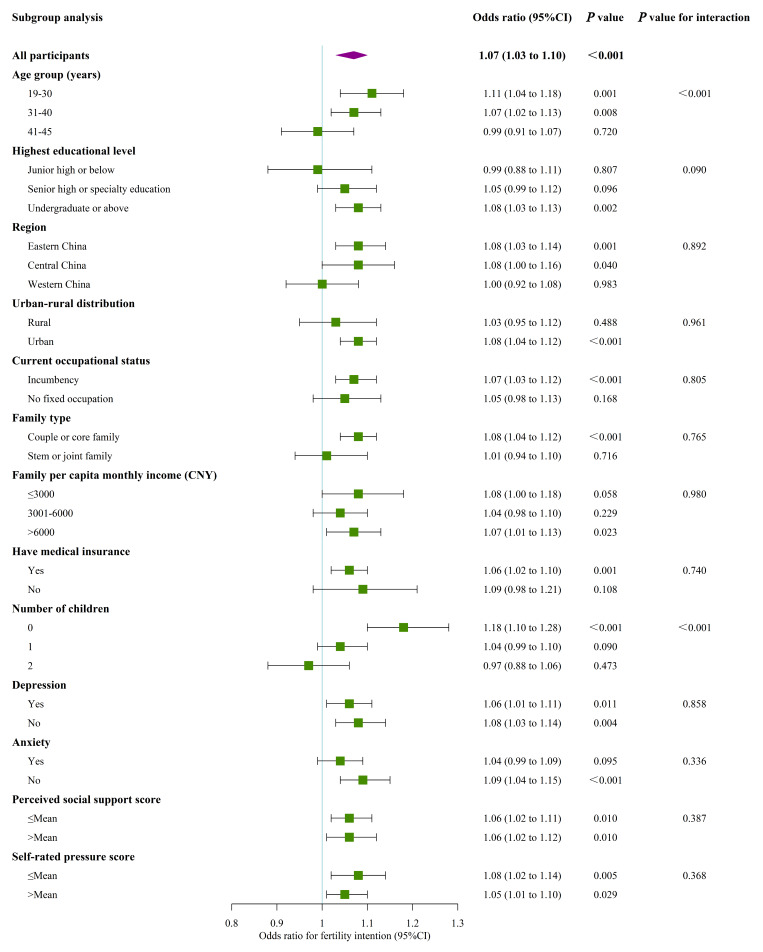
Subgroup analysis for the association between family health and fertility intention, with all covariates controlled except for subgroup variables. CI – confidence interval, CNY – Chinese Yuan.

The association between family health and fertility intention differed by age and number of children (**Figure 3**). A positive relationship was observed among women aged 19–40 years, but not among those aged 41–45 years. The association was most pronounced among women without children (OR = 1.18), while it was weaker and non-significant among women with one or more children, indicating effect modification by age and number of children.

## DISCUSSION

The results showed a positive linear association between family health and fertility intention, which was moderated by age and number of children. These findings highlight the importance of family health in promoting fertility intentions, particularly among younger women without children.

Our findings indicate that women embedded in families with higher overall health levels exhibit stronger fertility intentions, particularly regarding family social and emotional health, healthy family lifestyles, and external social support. Several factors may underpin this association. Strong family social and emotional health often reflects higher marital quality, effective communication, and cohesive family relationships. Such relational environments foster psychological security and shared reproductive goals, which have been shown to positively influence reproductive decision-making [36]. In addition, supportive marital relationships may buffer stress and promote emotional well-being, both of which are critical determinants of reproductive planning. Moreover, it has been shown that robust social support networks embolden childbearing aspirations [25,37]. Family external social support plays a pivotal role in reinforcing fertility intentions by providing normative encouragement, informational support, and emotional reassurance. Parental and family support has been empirically shown to be positively associated with fertility intentions, and this association operates partly through enhanced subjective well-being and reduced depressive symptoms, indicating psychological pathways through which social support may influence reproductive planning [38,39]. Individuals embedded in supportive social networks are more likely to express a willingness to have children [40]. That might be related to the fact that robust social ties can alleviate uncertainties related to pregnancy, childbirth, and parenting, thereby increasing women’s willingness to consider future fertility.

Psychological well-being constitutes another important mechanism linking family health and fertility intentions. Mental health, including levels of anxiety, subjective well-being, and family communication quality, has been found to influence fertility intention, with better psychological states associated with higher fertility motivation [41]. Healthier family environments tend to promote improved psychological outcomes, such as lower perceived stress and better family communication, which in turn are associated with elevated reproductive aspirations [40]. Women experiencing positive emotional climates within their families may thus perceive motherhood as more attainable and rewarding. Furthermore, broader social determinants of family healthy lifestyles, including access to community-level resources such as healthcare services, childcare facilities, and social welfare programs, are increasingly recognised as influential factors shaping fertility intentions by reducing barriers to childbearing and facilitating long-term reproductive planning [42].

It is also important to acknowledge the potential bidirectional relationship between family health and fertility intentions. Women with stronger fertility intentions may be more likely to perceive their family environment more positively, particularly regarding family emotional health and external social support, which could partially account for the observed associations.

Subgroup analysis showed no statistically significant association between family health and fertility intentions among women aged 41–45 years. This finding may indicate that, at later reproductive ages, factors other than family health play a more prominent role. First, advancing age is correlated with increased risks of infertility, pregnancy complications, and adverse pregnancy outcomes [43–45]. In women aged 41–45 years, these biological constraints often become the primary determinants of fertility intentions, overshadowing the influence of family health. Additionally, women in this age group are typically at a later reproductive stage [46], having often already achieved their desired family size, further limiting the impact of family health. Regarding the children-related impact of family health on fertility intentions, several points warrant consideration. First, the family’s economic situation significantly impacts reproductive decisions, especially for women with children [8,47]. Financial pressures related to further childbearing often outweigh the influence of family health. Moreover, shifting cultural attitudes from ‘more children, more happiness’ to ‘quality upbringing’ leads women to prioritise the development of existing children, reducing the influence of family health. Lastly, women with children may prioritise maintaining family stability over expansion, further diminishing the role of family health. However, these explanations lack direct empirical evidence and require further research for validation.

Overall, our findings indicate that improving family health is crucial for promoting fertility intention, although the magnitude of this association varies across population subgroups, underscoring the need for differentiated policy responses. For younger women without children, policy efforts should prioritise enhancing family social and emotional health and external social support. Government and community authorities could expand access to premarital and early-marriage family health services, including relationship education, mental health screening, and stress management programs, to support stable family formation and positive expectations regarding childbearing. At the community level, neighbourhood-based mutual support networks may further strengthen external social support, with community organisations and grassroots governance bodies facilitating peer support, family-oriented activities, and social cohesion. For women with children, whose fertility intentions appear less sensitive to family health, policy interventions should focus on alleviating structural constraints related to childcare and caregiving burdens. Local governments may expand affordable childcare services, improve parental leave policies, and strengthen intergenerational caregiving support systems to reduce the perceived costs of additional childbearing. Ultimately, a differentiated family health policy framework is warranted, one that tailors service content and delivery mechanisms according to women’s age, parity, and family life stage.

### Limitations

This study benefits from a large sample size, which enhances the validity and generalisability of the findings. Nevertheless, several limitations warrant consideration. First, the cross-sectional design precludes causal inference about the relationship between family health and fertility intentions. Second, self-selection effects cannot be excluded, as women with stronger fertility intentions may report more favourable perceptions of family health. Longitudinal studies or randomised controlled trials are needed to clarify causal pathways and underlying mechanisms and to minimise reporting bias. Third, adjustment for psychological and social variables that partially overlap with family health may have led to over-adjustment and attenuation of effect estimates; however, sensitivity analyses yielded directionally consistent results. Finally, family health and fertility intentions are multifaceted constructs influenced by numerous factors. Although major confounders were controlled, residual confounding from unmeasured variables may persist. Future research should incorporate additional high-impact indicators, including relationship quality, family economic insecurity, childcare availability, intergenerational cohabitation, and mental health status, to further elucidate this relationship.

## CONCLUSIONS

Our study identified a positive linear association between family health and fertility intention among Chinese women of childbearing age, with particularly strong effects among younger nulliparous women. These findings underscore family health as a promising intervention target for improving fertility intention. Tailored interventions that enhance family health, especially during early reproductive years, may be more effective when aligned with women’s family life stages. Future longitudinal studies incorporating comprehensive measures of family dynamics are needed to clarify causal mechanisms, while policymakers may consider embedding family health-focused strategies within multifaceted frameworks to address persistently low fertility rates.

## Additional material


Online Supplementary Document
